# The African Goat Improvement Network: a scientific group empowering smallholder farmers

**DOI:** 10.3389/fgene.2023.1183240

**Published:** 2023-08-29

**Authors:** Curtis P. Van Tassell, Benjamin D. Rosen, M. Jennifer Woodward-Greene, Jeffrey T. Silverstein, Heather J. Huson, Johann Sölkner, Paul Boettcher, Max F. Rothschild, Gábor Mészáros, Helen N. Nakimbugwe, Timothy N. Gondwe, Farai C. Muchadeyi, Wilson Nandolo, Henry A. Mulindwa, Liveness J. Banda, Wilson Kaumbata, Tesfaye Getachew, Aynalem Haile, Albert Soudre, Dominique Ouédraogo, Barbara A. Rischkowsky, Ally Okeyo Mwai, Edgar Farai Dzomba, Oyekanmi Nash, Solomon Abegaz, Clet Wandui Masiga, Maria Wurzinger, Brian L. Sayre, Alessandra Stella, Gwenola Tosser-Klopp, Tad S. Sonstegard

**Affiliations:** ^1^ Animal Genomics and Improvement Laboratory, USDA Agricultural Research Service, Beltsville, MD, United States; ^2^ National Agricultural Library, USDA Agricultural Research Service, Beltsville, MD, United States; ^3^ Office of National Programs, USDA Agricultural Research Service, Beltsville, MD, United States; ^4^ Department of Animal Science, Cornell University, Ithaca, NY, United States; ^5^ Division of Livestock Sciences, University of Natural Resources and Life Sciences, Vienna, Austria; ^6^ Animal Production and Health Division, Food and Agriculture Organization of the United Nations, Rome, Italy; ^7^ Department of Animal Science, Iowa State University, Ames, IA, United States; ^8^ National Animal Genetic Resources Centre and Data Bank, Entebbe, Uganda; ^9^ Department of Animal Science, Lilongwe University of Agriculture and Natural Resources, Lilongwe, Malawi; ^10^ Biotechnology Platform, Agricultural Research Council, Pretoria, South Africa; ^11^ National Agricultural Research Organization, Entebbe, Uganda; ^12^ International Center for Agricultural Research in the Dry Areas, Addis Ababa, Ethiopia; ^13^ Unité de Formation et de Recherches - Sciences et Technologies, Université Norbert ZONGO, Koudougou, Burkina Faso; ^14^ Université Joseph Ki-Zerbo, Ouagadougou, Burkina Faso; ^15^ International Livestock Research Institute, Nairobi, Kenya; ^16^ Discipline of Genetics, School of Life Sciences, University of KwaZulu-Natal, Pietermaritzburg, South Africa; ^17^ National Biotechnology Development Agency, Abuja, Nigeria; ^18^ Ethiopian Institute of Agricultural Research, Addis Ababa, Ethiopia; ^19^ Tropical Institute of Development Innovations (TRIDI), Mukono, Uganda; ^20^ Department of Biology, Virginia State University, Petersburg, VA, United States; ^21^ Institute of Agricultural Biology and Biotechnology (IBBA), Milano, Italy; ^22^ GenPhySE, Université de Toulouse, INRAE, ENVT, Castanet Tolosan, France; ^23^ Acceligen Inc., Eagan, MN, United States

**Keywords:** goats, genomics, genetics, community-based breeding programs, sustainability, small-holder

## Abstract

The African Goat Improvement Network (AGIN) is a collaborative group of scientists focused on genetic improvement of goats in small holder communities across the African continent. The group emerged from a series of workshops focused on enhancing goat productivity and sustainability. Discussions began in 2011 at the inaugural workshop held in Nairobi, Kenya. The goals of this diverse group were to: improve indigenous goat production in Africa; characterize existing goat populations and to facilitate germplasm preservation where appropriate; and to genomic approaches to better understand adaptation. The long-term goal was to develop cost-effective strategies to apply genomics to improve productivity of small holder farmers without sacrificing adaptation. Genome-wide information on genetic variation enabled genetic diversity studies, facilitated improved germplasm preservation decisions, and provided information necessary to initiate large scale genetic improvement programs. These improvements were partially implemented through a series of community-based breeding programs that engaged and empowered local small farmers, especially women, to promote sustainability of the production system. As with many international collaborative efforts, the AGIN work serves as a platform for human capacity development. This paper chronicles the evolution of the collaborative approach leading to the current AGIN organization and describes how it builds capacity for sustained research and development long after the initial program funds are gone. It is unique in its effectiveness for simultaneous, multi-level capacity building for researchers, students, farmers and communities, and local and regional government officials. The positive impact of AGIN capacity building has been felt by participants from developing, as well as developed country partners.

## Graphical Abstract

**Figure:**
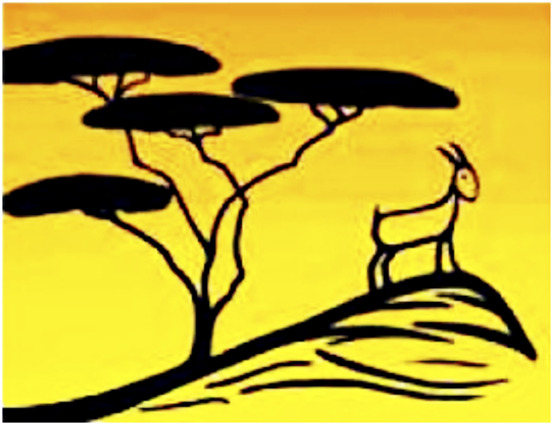

## 1 Introduction

### 1.1 Background

**FIGURE 1:**
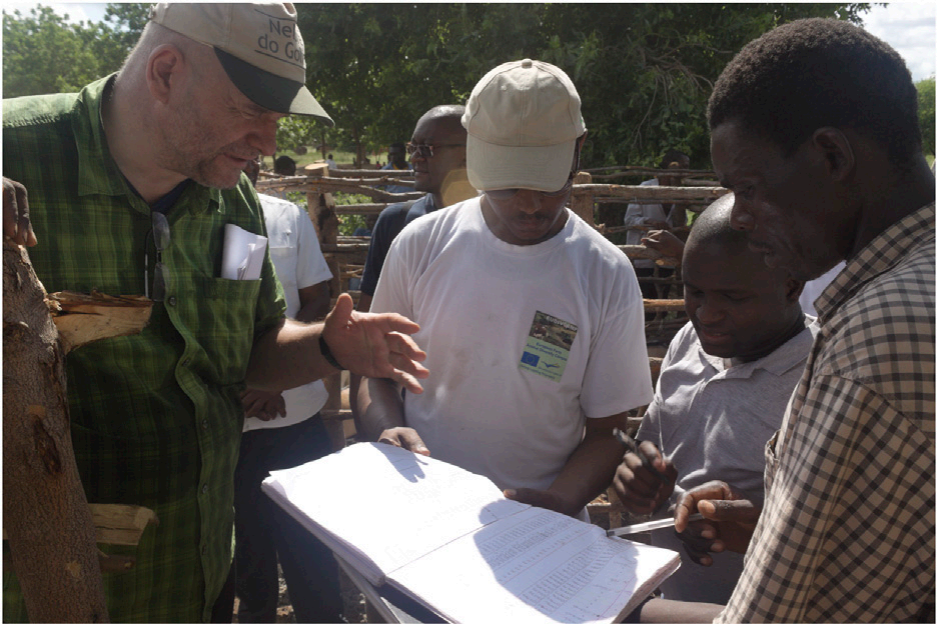
AGIN scientists and partners consider data recorded on goat growth rates.

Goats are crucial sources of milk, meat, and income for many smallholders in sub-Saharan Africa (Panin and Mahabile, 1997; Kosgey et al., 2008). Livestock are particularly critical to the poor in marginal areas where crop yields are inadequate and ruminants can convert low-quality feedstuffs into high-quality dietary protein for humans (McDowell, 1988). Goats have been naturally selected as well as selectively bred (Figure 1) to accommodate the highly variable conditions across sub-Saharan Africa resulting in locally adapted populations (Daramola and Adeloye, 2009; Karnuah et al., 2018). Goats have several advantages, particularly over cattle, that allow them to contribute to socio-economic development of Africa. Significantly, goats browse and can consume a wide range of grasses, leaves, and feeds that people find unappealing or are unable to digest. Additionally, goats have the ability to travel great distances in search of feed and have a small body size resulting in reduced feed requirements. Finally, goats have high reproductive rates (i.e., multiple births) and short generation intervals. Geographic isolation and genetic bottlenecks of goats in African populations have yielded a vast resource of phenotypic and genetic variation within and among native breeds.

### 1.2 Problem

Despite this tremendous genetic resource, large portions of sub-Saharan Africa remain food insecure (Smith et al., 2006). The simplicity of the explanation of that problem belies the complexity of a solution. The lack of productivity lies at the intersection of basic practices: traditional animal husbandry to manage production systems, pedigree and performance recording, and selective breeding. Much of the deficits seen in these production systems are best met with an outreach program very much like the cooperative extension system that had such a huge impact in transferring improved agricultural practices and technologies in the U.S. over the 20th century (Rasmussen, 1989).

### 1.3 Community-based breeding programs

To facilitate this knowledge transfer in the absence of a formal cooperative extension service, a system of community-based breeding programs (CBBP; see Abbreviations Table (Supplemental Materials)) could leverage local researchers and technical experts familiar with the traditional animal husbandry production systems of that community. CBBPs have recently grown in popularity (Wurzinger et al., 2021). With a CBBP approach, farmers and local communities actively participate in the decision-making using their priorities and preferences. This strategy is usually built on locally-adapted and indigenous breeds of livestock with a goal of sustainable intensification. Genetic improvement is increased in most CBBP as opposed to farmers selling the best (i.e., heaviest) offspring, which would induce a negative selection (Gizaw et al., 2014; Haile et al., 2018).

### 1.4 Research goals

The initial goals of our efforts combined science and application. The scientific component of this project involved two distinct efforts. The first was the sampling of African goat breeds and populations followed by genomic characterization to better understand genetic diversity and within and across population variation of the African goats. Once the existing variation was characterized, a framework could be established for migration and admixture between those populations. The second scientific goal was the identification, description, and use of “signatures of selection.” Selection signatures are genomic footprints that provide evidence of historic selection (Kreitman, 2000; de Simoni Gouveia et al., 2014). The failure of non-adapted goats created from advanced backcross or intercross populations to thrive when exposed to extensive natural conditions and the related stressful environments in Africa is compelling evidence of the genetic component to adaptation (Hassen et al., 2002; Tibbo, 2006; Escareño Sánchez, 2010). The practical components of this project involve outreach, capacity building, and technology transfer. We believe that the most important practical component is the application of CBBPs (Mueller et al., 2015), a tool that is key to sustainability.

## 2 AGIN: The African Goat Improvement Network

### 2.1 AGIN overview

The first workshop was held in Nairobi, Kenya in 2011 and continued through the re-branded AGIN II meeting in 2013 (Entebbe, Uganda), AGIN III in 2014 (Addis Ababa, Ethiopia), AGIN IV in 2016 (at FAO in Rome, Italy), and AGIN V in 2017 (Pretoria, South Africa). The goals of this diverse group were to: improve indigenous goat production in Africa; characterize existing goat populations and to facilitate germplasm preservation where appropriate; and to combine the use of genomics to understand adaptation. The long-term goal was to develop cost-effective strategies to improve productivity of small holder farmers without sacrificing adaptation.

### 2.2 The beginning—Events leading up to the first meeting

In the time leading up to the initiation of the project that spawned the AGIN group, the BovineSNP50 genotyping tool (Matukumalli et al., 2009) was beginning to have impact on dairy cattle genetic improvement through the application of genome selection (Meuwissen et al., 2001; VanRaden et al., 2009; Garcia-Ruiz et al., 2016) in the US. The tools being developed at that time were mostly based on single nucleotide polymorphisms (SNP), or single base changes, in the DNA sequence. In addition to the use of SNPs to implement genome selection, these SNPs were being used for verification of parentage or even to discover putative parents for animals with unknown or incorrect parentage (Gibbs et al., 2009). The initial idea of the United States Department of Agriculture (USDA)—Agricultural Research Service (ARS) research group was to simplify the genome selection strategy, but still apply genomics to genetic improvement in goat populations in Africa. This assumption proved to be wildly overly simplistic.

#### 2.2.1 Serendipity strikes

**FIGURE 2:**
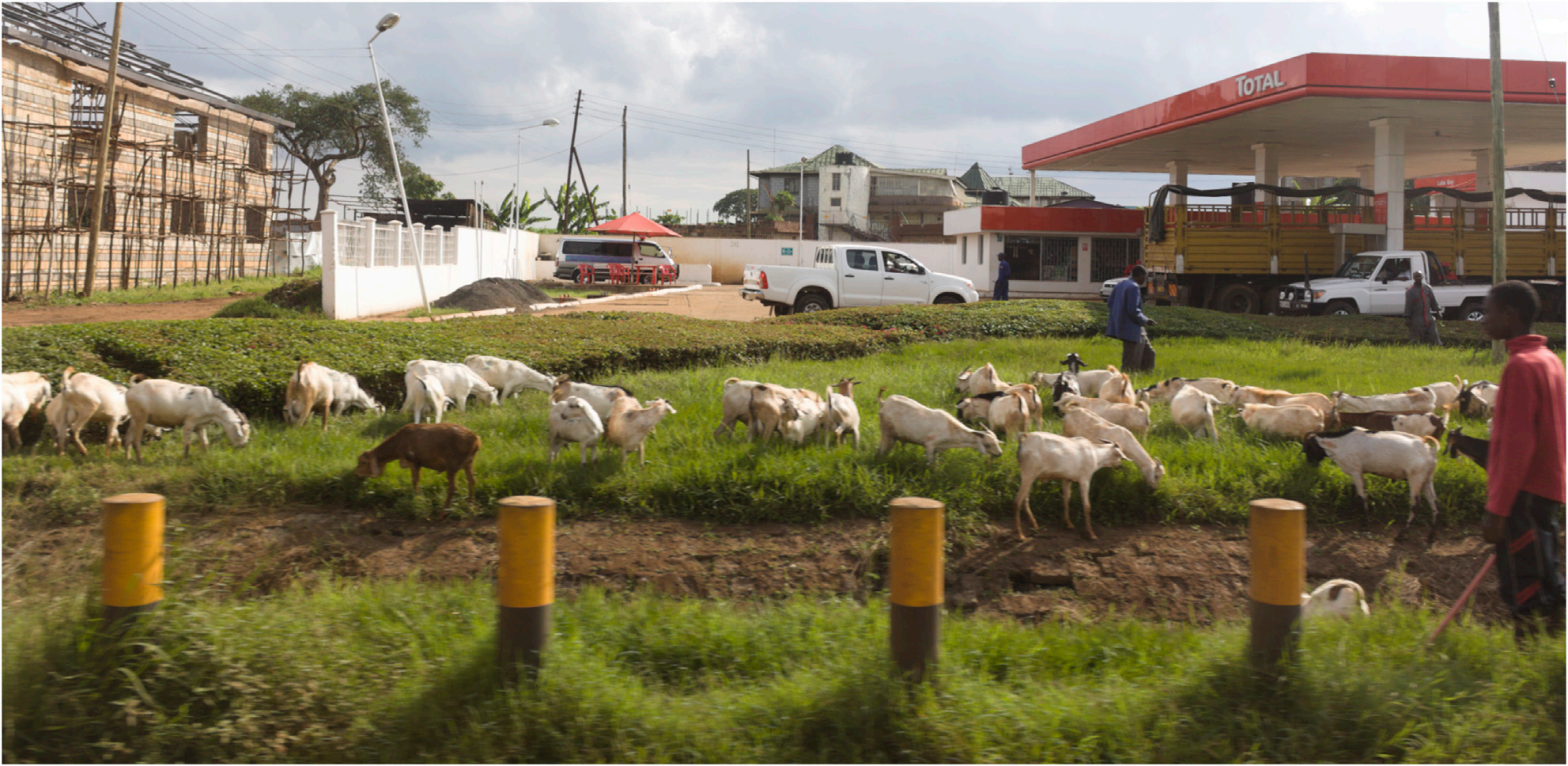
Goats roaming the streets of Nairobi.

There were several events that were serendipitous despite being quite important in the process of forming the project that eventually led to the AGIN group. One of the first of these events was the invitation of one of the ARS scientists (CPVT) to a livestock genetics expert consultation meeting held in Nairobi, Kenya sponsored by the Bill and Melinda Gates Foundation (BMGF) in February 2009. An afternoon excursion during that visit to several neighborhoods around Nairobi made clear that indigenous goats were thriving in this environment (Figure 2). Understanding the genetics and biology of adaptation has been a high priority of this project since that moment. The importance of goats as a source of high-quality protein and as a repository of assets became apparent during that tour of Nairobi. Several discussions about the importance of goats in small holder production systems during the BMGF meeting confirmed and even enhanced that observation.

At the same time another member of the ARS team (JTS) spent 3 months fostering collaboration between USDA and the Bureau of Food Security at the US Agency for International Development (USAID). During that time, the US government’s global hunger and food security initiative, Feed the Future, introduced the Norman Borlaug Commemorative Research Initiative, a collaborative research effort between ARS and USAID. The project, “*Improving Livestock Productivity through Enhanced Breeding Programs,*” was funded through this initiative and began immediately with the first meeting of the group that would become the AGIN consortium in the fall of 2011 at the International Livestock Research Institute (ILRI) in Nairobi. The project is typically just called “*The Goat Improvement Project.*”

#### 2.2.2 Initial objectives

The initial project proposal submitted to USAID contained four primary objectives. First, to sequence and build a *de novo* assembly of the domestic goat genome and to discover a large number of SNP markers to enable construction of a high-density genotyping array. Second, to conduct a workshop to enlist partners and establish a strategy for developing and deploying genomic and genetic tools. Third, to genotype 15 individuals per breed at high density (50K) for 50 breeds for a total of 750 animals. Finally, to genotype 2000 individuals at reduced density, collect phenotypes from those animals, and establish a training and outreach network. There were ongoing efforts led by the International Goat Genome Consortium (IGGC) to build a goat genome assembly (Dong et al., 2013) and to develop a high-density genotyping platform, the GoatSNP50 chip (Tosser-Klopp et al., 2014). The AGIN project took advantage of the international efforts, despite uncertainty that these tools would be well-adapted to African goat studies.

### 2.3 The formation of AGIN—The African Goat Improvement Network

The central aim of the goat improvement project was to catalyze a regional, perhaps even continental, cooperative effort to apply genomic tools to aid characterization of the structure of caprine genomes in locally-adapted, native breeds throughout sub-Saharan Africa. Using this approach, the intention was to develop genomic tools for animal improvement efforts in Africa. There was also a strong desire to establish regional cooperation so that an individual country breeding program could leverage the efforts and collective expertise of the group members. In other words, to provide a nexus to enhance the cooperative efforts of advanced research institutions, such as ARS, ILRI, and colleges and universities.

This project deployed a unique, three-pronged approach to livestock improvement in the developing world, especially in Africa. First, the project focused on long-term, sustainable solutions by bringing together classical breeding programs and fundamental animal husbandry techniques as prerequisites to implementing genomic-based approaches. Second, the ARS research group has focused attention on development of partnerships with established research and outreach programs in the specific countries that we targeted. The third feature of the AGIN approach, was to integrate opportunities for capacity building throughout the program at all levels of implementation, including farmers, students, researchers, and government and policy-making officials. The AGIN brought together top experts working in African developing communities and directly engaging farmers. Through these efforts the group worked to make state of the art technology more accessible to African small holders, researchers, and government officials concerned with animal genetic improvement and conservation. The AGIN group also recognized the importance of including social scientists and economists in the project to maximize market opportunities for goats and to document the impact of goats on the livelihood of small holders in Africa.

### 2.4 AGIN I—The beginning

#### 2.4.1 Field visit

**FIGURE 3:**
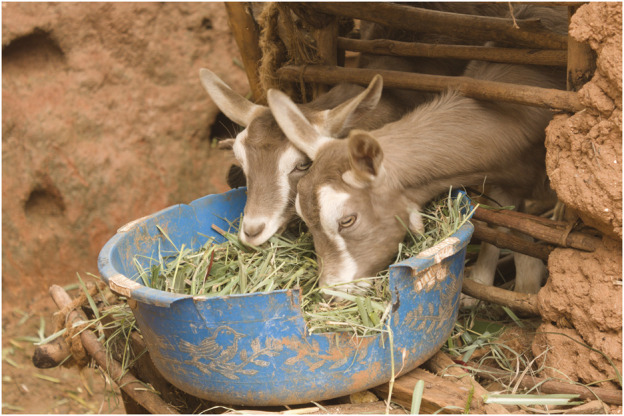
Well managed goats observed on the AGIN I field visit.

From the start, the AGIN meetings have been composed of two distinct elements. The first component has been field visits to interact with producers or other members of the goat value chain. The field visit associated with the first group meeting was a trip to the Mwingi district of Kenya (approximately 150–200 km northeast of Nairobi) on November 28 and 29, 2011. The purpose of the field trip was to view the array of smallholder goat production systems in that area, understanding that this location was but one region of Africa with a subset of farming and agribusiness practices. Participants in the field trip represented a variety of governmental and non-governmental agencies, research groups, and universities. The 2-day field trip was led by personnel from Farm Africa, a non-governmental organization working on livestock improvement in East Africa. The group visited a number of facilities, including a pastoral goat production system, an auction market, community-based production systems (Figure 3), breeding stations, and a purebred dairy goat operation. Each visit provided an opportunity to discuss with the farmers, mostly women and generally small holders, the impact of generating meat, milk, or revenue had in their lives or their families. The group also discussed the challenges and opportunities the small holders had in marketing their products.

#### 2.4.2 Workshop

The second phase of each AGIN gathering has been highlighted by a workshop incorporating new observations from the recent field visit, description of production systems and practices in counties represented at the workshop, and presentations from experts across goat research areas. From the beginning, we have emphasized the importance of discussions and fostered diversity of opinions among participants. The model—visit with local goat smallholders and then convene a workshop to discuss the observations of the group and evaluate assumptions—is to observe well managed production systems and then do a reality check on the aims and approaches of the group. The meeting *“Workshop I: Defining technical aspects of sequencing the goat genome, outlining project goals”* was conducted on November 30 and December 1, 2011 and included 23 participants. This first meeting was dominated by delegates from Kenya and the U.S., with nine and five attendees, respectively. The remaining participants represented Austria (2), Brazil (1), China (1), Syria (1), Tanzania (1), Uganda (2), and the U.K. (1).

There were a number of observations made during that initial workshop. From the field visits, it was apparent that large-scale on-farm data collection or tissue sampling to enable DNA extraction and eventual genotyping was impractical. The application of genomic tools in large scale was also deemed unrealistic. The group also concluded that wholesale replacement of indigenous goats with those selected for production in temperate climates had been largely unsuccessful, as many groups have observed (Kosgey et al., 2006). Furthermore, the workshop attendees felt that the locally-adapted goats were an important resource that should be more comprehensively characterized and the biology of adaptation, in particular, needed to be better understood. The assembled group was very supportive of the training provided by Farm Africa that accompanied the introduction of elite breeding stock. The farming practices learned by these program participants enhanced productivity through improved animal health, nutrition, and reproduction. It was noted, however, that in many cases alternative methods are needed for application of technology by smallholders. Finally, there was nearly universal support for ARS scientists to continue development of a high-quality genome assembly for the goat.

At the close of the meeting, the objectives of the goat improvement project were changed substantially. The first two objectives were largely intact from the original proposal, specifically, first, to conduct workshops that brought together a broad range of people with an interest in sustainable, locally-adapted goat production systems and, second, to develop a true *de novo* assembly of the caprine genome, as we focused on improving the existing assembly. The third set of goals was to characterize indigenous goats of African smallholders. This set of goals included identifying goat populations to characterize, collecting samples, extracting DNA, generating genotype and sequence data, and conducting analyses to identify signatures of selection.

We eventually concluded that the GoatSNP50 performed well when genotyping African goats, and it became clear that it was unnecessary to designing a new SNP-chip. Instead, the AGIN partnered with the IGGC to contribute design efforts to upcoming versions of the chip. The ultimate objective of this goal was to identify population-based signatures of selection with genotypic data along with data from targeted resequencing of these adapted populations. These signatures could then be traced with data from low-density SNP panels across generations to ensure that those areas previously impacted by selection would be maintained while introgressing loci elsewhere in the genome. The assays would provide an inexpensive test of breeding animals, allowing for enhanced productivity while maintaining adaptation and fitness in the existing production system. The expectation driving this approach is that this strategy would increase productivity while maintaining genetic variation in the indigenous goat population. An additional goal was identified—create a name for the group that was coalescing. Following this meeting the name African Goat Improvement Network—AGIN was agreed on by the group.

In January 2012 the project and one of the PIs (TSS) received the Illumina Agricultural Greater Good Initiative Award, including 400 GoatSNP50 genotyping assays and discount on any additional goat assays supporting this project. This award allowed us to significantly increase the scope of the project. In addition, Egyptian samples were collected and genotyped through support of the Greater Good Initiative.

### 2.5 AGIN II—Training for phenotype and tissue collection

#### 2.5.1 Workshop

The second workshop was hosted by the Association for Strengthening Agricultural Research in Eastern and Central Africa (ASARECA) in Entebbe, Uganda on March 12 and 13, 2013 and included 34 participants, and was again dominated by delegates from the host country, Uganda, and the U.S., with nine and 11 participants, respectively. The remaining participants represented Austria (2), Italy (2), Kenya (3), Malawi (1), Mozambique (1), Nigeria (1), South Africa (1), Tanzania (1), the U.K. (1), and Zimbabwe (1). At this meeting the workshop was held prior to the field visits. The workshop focused on the development of the AGIN. Additionally, the research group focused on the development of CBBP to create a sustainable environment for genetic improvement and information exchange. The members of the AGIN at that time represented 10 African universities and 3 regional research institutes.

During the AGIN workshop, several committees were developed to establish clear guidelines and expectations and to facilitate candid communications. These committees were intended to address:

1) guidelines for collaboration, including publications and authorship, data access, funding recognition, and access to materials and data;
2) animal sampling prioritization and logistics;
3) phenotype collection;
4) genetic resources and conservation; and
5) outreach education and training.

#### 2.5.2 Field visits

**FIGURE 4:**
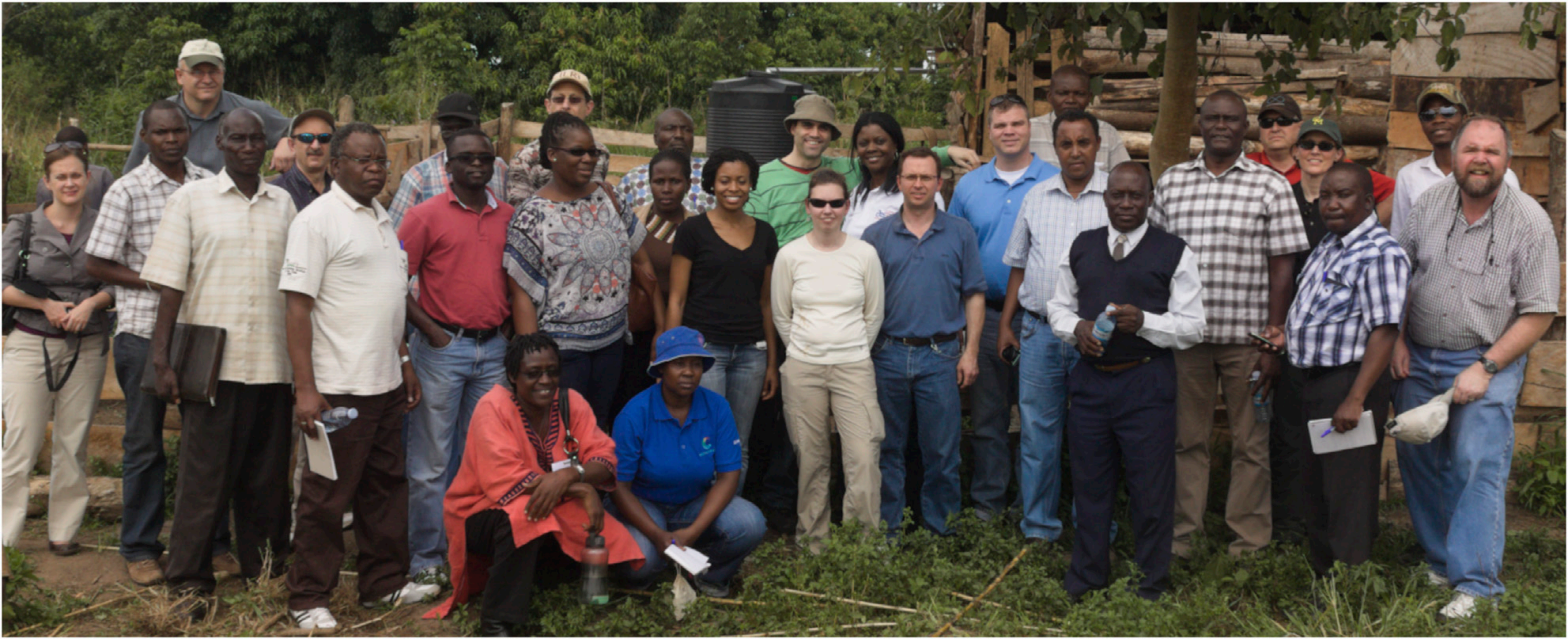
Members of AGIN II gathering in Uganda.

Five farm visits took place from Kampala on 14 March 2013 to Luweero and Wakiso districts and March 15 to Mukono and Jinja (Figure 4). The participants discussed the AGIN project goals with farmers, who represented a broad range of resource constrained production systems. Goat herd sizes ranged from 2 goats to several hundred, and production systems ranged from highly extensive to intensively managed.

A large number of AGIN members were trained to collect phenotypes and tissue sampling using a standardized method named the AGIN image collection protocol (AGIN-ICP). Researchers from Ethiopia, Italy, Kenya, Malawi, Mozambique, Nigeria, Rwanda, South Africa, Tanzania, Uganda, The United States, and Zimbabwe were trained to obtain digital images and to collect body measures. Coordination of sample collection was led by ASARECA and ARS. At that time, phenotypes (digital images and body measurements) and tissue samples were collected from more than 1,800 goats in 10 countries (7 African countries).

### 2.6 AGIN III—Focus on community-based breeding programs (CBBP)

The International Livestock Research Institute (ILRI) in partnership with ARS co-organized AGIN III, with a workshop entitled “*Best Practices for Community-Based Breeding Programs (CBBP) - Genetic Improvement of Goats.*” The meetings were held on June 12–13, 2014, in Addis Ababa, Ethiopia. Attendees included individual farmers and CBBP implementers, representatives from universities and research organizations, as well as government ministries (USDA-ARS, USAID, ILRI, International Center for Agricultural Research in the Dry Areas (ICARDA), ASARECA, Food and Agriculture Organization of the United Nations (FAO), and Embrapa) representing 16 countries (11 African countries (Ethiopia, Kenya, Malawi, South Africa, and Uganda) and Australia, Austria, Brazil, Italy, and the US).

#### 2.6.1 Field visits

**FIGURE 5:**
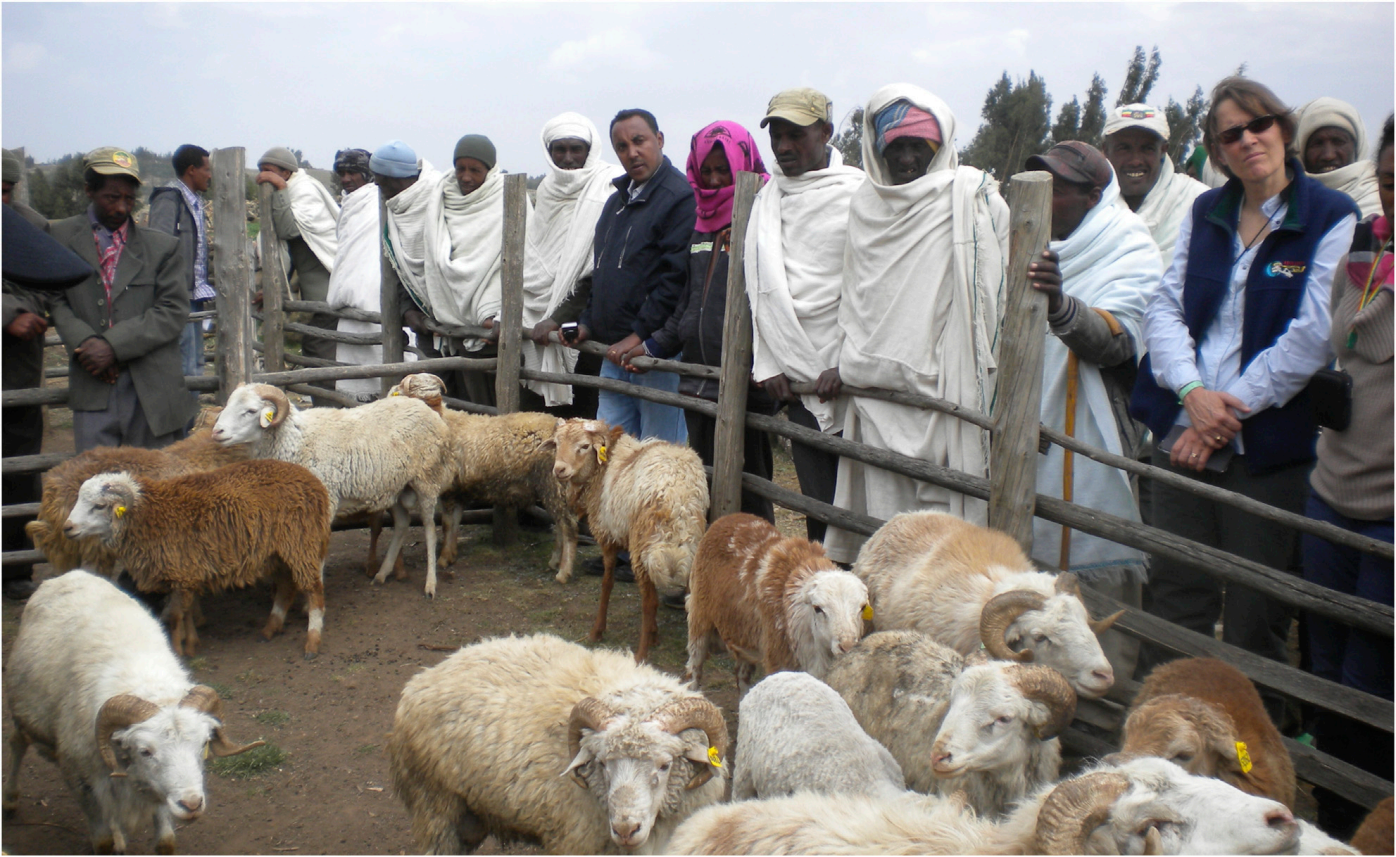
Members of AGIN visited a community-based breeding program in the Mentz region of Ethiopia and observed an annual selection of the best sheep in the collective flock as part of the AGIN III field visit.

A group of about 20 AGIN III workshop attendees participated in a 2-day field trip held before the workshop on June 10–11, 2014. The purpose of the tour was to visit sheep CBBP in the villages of Molale and Mehal Meda in Menz, Ethiopia (Figure 5). Visits to the Menz communities provided highly successful examples of CBBP and offered a valuable opportunity to see collaborative efforts in action. These visits also gave AGIN partners an opportunity to interact directly with smallholders and learn their views of the CBBP. Most importantly, these visits to CBBP demonstrated the impact of the projects on the lives of the participants. The two CBBP that were visited were established in 2008. Researchers worked with villagers to determine their breeding goals, and ram selection based on these goals began in 2010. The project included about 60 households participating in each village. The formation of these CBBP was led by Johann Sölkner, an active member of AGIN.

Several Ethiopian graduate students earned their doctoral degrees conducting research on these CBBP under the direction of Professor Sölkner. These newly trained researchers joined AGIN and were active participants, sharing their knowledge and experiences with the communities. One of the principal objectives of AGIN is to foster the development of local capacity that will form a nucleus of expertise for African CBBP in the future. These African students also will provide leadership and invaluable guidance to the overall AGIN CBBP efforts across the continent.

Participants in the field trip observed the CBBP in action, as the farmers conducted a selection for the best ram lambs. There was also a competition for the best young rams and ewes. Awards were sponsored by the USAID Feed the Future Initiative, with ribbons provided as recognition for the best animals. At the close of the ceremony, the smallholders addressed the group. They thanked the researchers who had worked with them over the years to develop the CBBP and explained that their animals were now known as a high-quality product and commanded a higher price in the markets. They also expressed intense appreciation for the improvement seen in their flock, as evidenced by their animals’ enhanced ability to cope with current drought conditions that have caused food shortages for animals in other villages in the area. The villagers described their recent achievement in gaining legal status as a cooperative, giving them the ability to apply to aid organizations for veterinary services and other benefits.

#### 2.6.2 Workshop

Based on the observations on the field visits, the workshop discussion focused largely on determining best practices for CBBP implementation for sustainable small holder goat breeding programs. This topic was well aligned with several AGIN project objectives and was timely with AGIN CBBP activities being initiated in Uganda and Malawi in 2014. In addition, there were reports updating AGIN members on current research projects and future directions. Specific updates included the *de novo* genome assembly of the domestic goat, genetic characterization of indigenous, exotic, and admixed populations, development and analyses of a digital phenotype collection, analysis of body size variation and finally, a report on consideration of the Boer breed that originated in South Africa and has spread across the African continent and the globe. Global comparisons were planned to be done with US (Spanish derived), New Zealand (Boer), Turkey (domestication center), Brazil (climate, parasite resistance) and Italy (dairy breeds) goats to find important adaptive traits present in African goat breeds. These traits were targets for acceleration of genetic improvement.

### 2.7 Between AGIN III and IV—New goals identified

With an increased focus on CBBP, establishing CBBP was added as an official project goal and efforts were divided into 4 sub-goals. The overarching goal was to establish CBBP for small holder goat producers. The four sub-goals added were:

1. To establish in country scientific partnerships and to identify communities to host CBBP;
2. To select founding stock and initiate breeding programs;
3. To genotype and analyze founder animals developing smallholder DNA tools as needed; and,
4. To benchmark genetic progress of these CBBP.

### 2.8 AGIN IV—Implementation of community-based breeding programs (CBBP)—“It takes a village…”

The AGIN efforts were designed to bring smallholders located in developing economies into the 21st century as full players and partners. As of 2016, the AGIN community represented nearly 40 research, educational, or international development institutions from 20 countries, 12 of them African. To reduce the travel costs of the combined events, the format of the AGIN IV meeting was altered. The field visits were made to two of the Malawi CBBP just prior to the workshop, which was held in Rome, Italy at the headquarters of the FAO.

#### 2.8.1 Field visit

**FIGURE 6:**
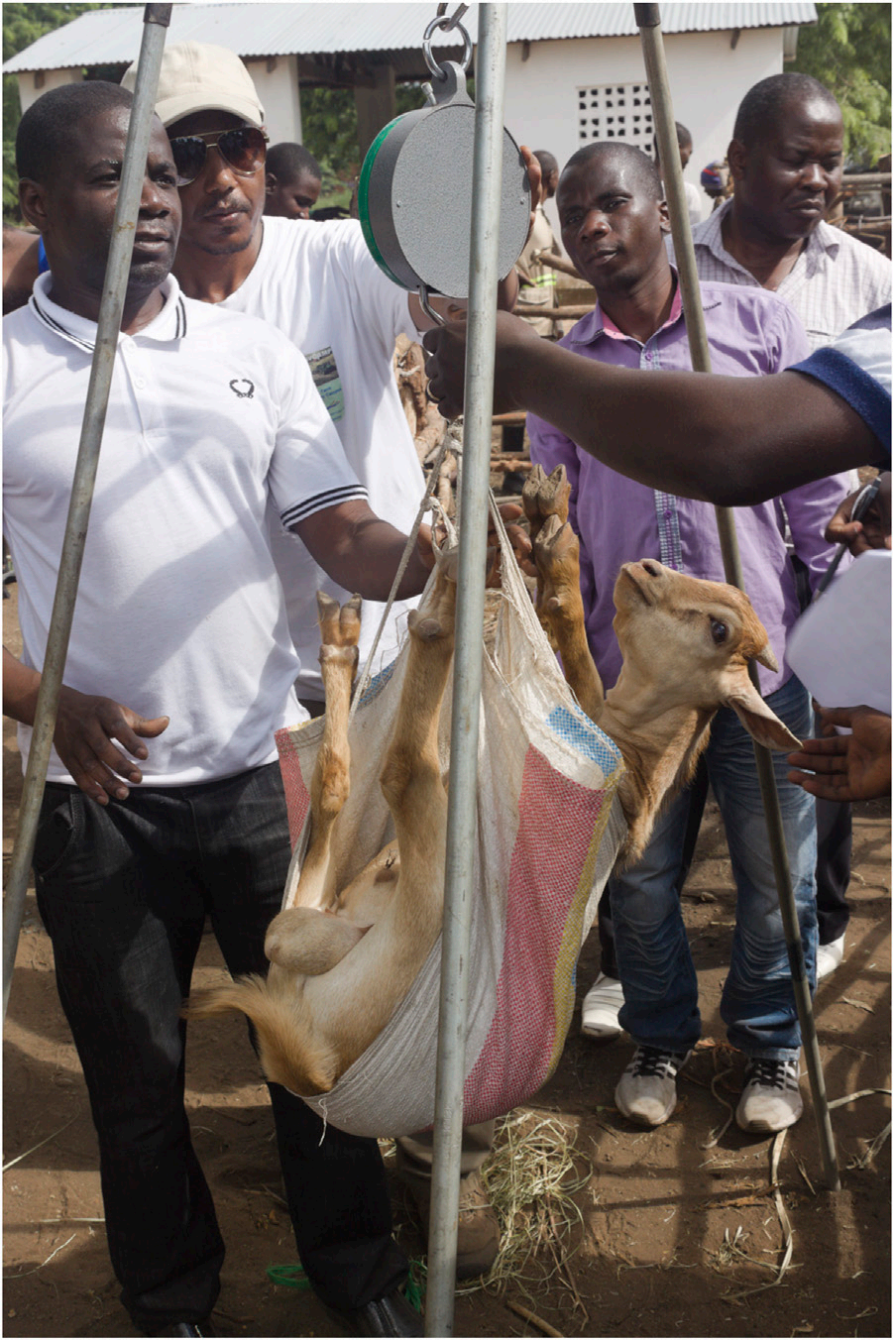
AGIN member participate with community selection process as part of the AGIN IV field visit.

A relatively small team visited two of four CBBP sites in Lower Shire, Malawi. The CBBP collaboratively developed breeding goals directly with small-holder farmers and designed a program to implement those goals (Figure 6). These efforts were funded by a collaborative research effort between USDA-ARS and USAID and facilitated by AGIN, a group of livestock, genetic, and international development experts. The AGIN model is a novel approach to build sustainable livestock improvement in developing countries by integrating direct input and training of farmers, extension, genetics, livestock and international development experts. The ultimate goal was to build sustainable animal genetic improvement to enhance human, livestock and economic health in the community. Also attending the Malawi site visits were Ugandan, South African, and Austrian project partners, and the regional Program Manager and staff of the Shire Valley Agricultural Development Division of the Malawi Ministry of Agriculture, Irrigation and Water Development.

#### 2.8.2 Workshop

**FIGURE 7:**
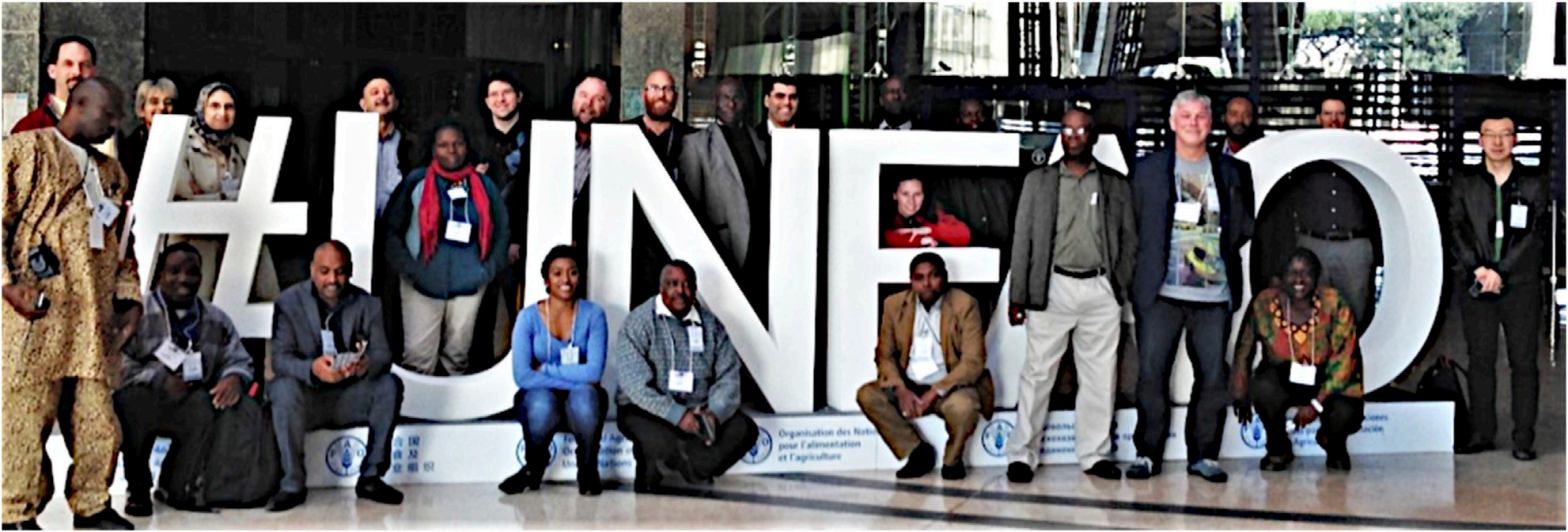
Members of AGIN IV workshop at the United Nations Food and Agriculture Organization (UNFAO) in Rome, Italy.

The AGIN IV workshop was hosted at the FAO headquarters in Rome, Italy on February 22–24, 2016. A total of 43 participants from 17 countries, representing government and university researchers, international development experts, post docs, and graduate students attended, including representatives from USAID in Washington, DC and the US mission in Rome (Figure 7). Specific outcomes included a draft strategic plan to implement, test, and evaluate a novel approach to livestock development focused on long-term, sustainable solutions via integration of 1) community-based breeding programs (CBBP), 2) application of modern genomics and genetic tools based on farmer input for use within the CBBP, and 3) multi-level networking and capacity building. Much of the discussion at the workshop focused on the limited time remaining for funding to continue from USAID and USDA-ARS and developing a continuity strategy for the funded projects to establish a plan to ensure sustainability.

### 2.9 AGIN V—The last waltz

**FIGURE 8:**
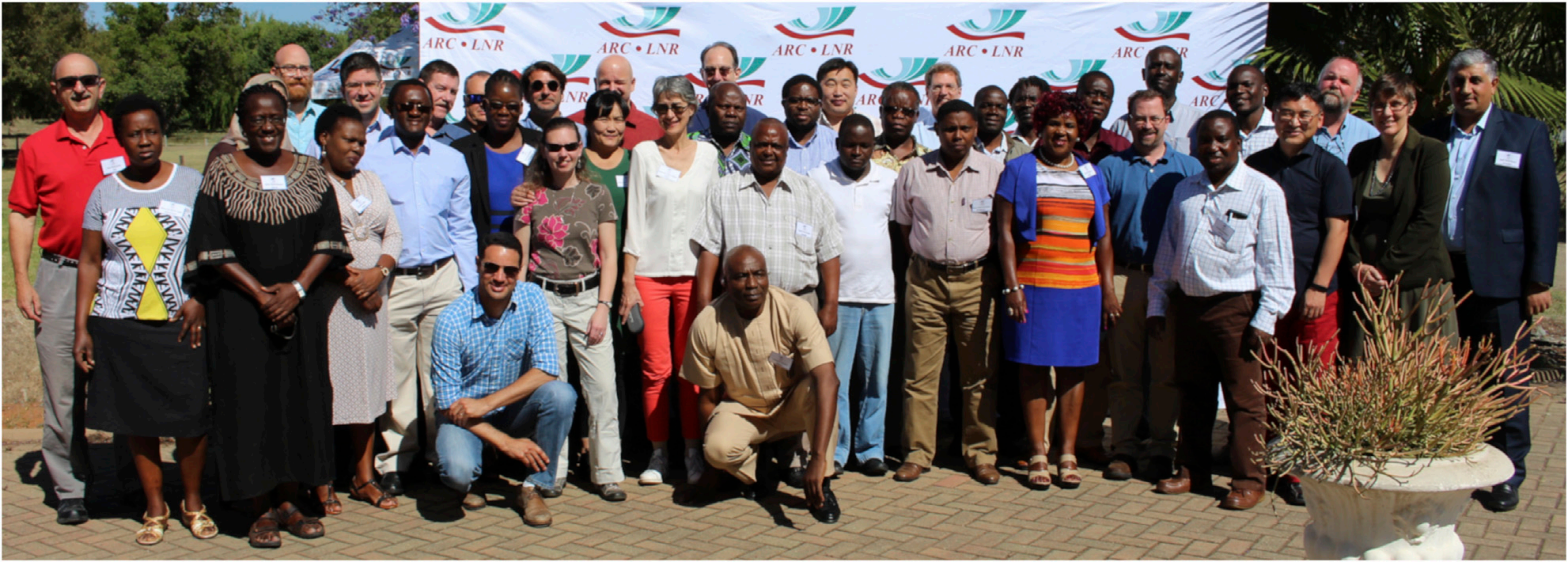
Members of the African Goat Improvement Network (AGIN) team at the AGIN V workshop.

The South African Agricultural Research Council (ARC) hosted the final AGIN (V) meeting. The meeting was held October 31 to November 2, 2017 at ARC facilities in Pretoria, South Africa. The goat improvement project funded travel and housing for 20 participants to attend this meeting. The Food and Agriculture of the United Nations (FAO) continued to collaboratively support the efforts of the project, and FAO funded 7 additional attendees. The AGIN V meeting was attended by over 40 participants, representing nearly 30 organizations from almost 20 countries (Figure 8).

#### 2.9.1 Site visits

The AGIN V meeting was preceded by a visit to an ARC sponsored CBBP in the village of Pella, North-West Province, South Africa on October 30, 2017. The Pella CBBP site visit coincided with a meeting of the village royal family, the local government board, and representatives of farmer organizations. Representatives of the AGIN group that travelled to Pella met with the local board and the “Kgosi,” or chief of the village, separately, and an informational meeting was led by representatives of ARC. In addition to meeting with these community members, the AGIN group visited two of the community farms and met with these producers.

#### 2.9.2 Workshop

The AGIN V workshop featured research updates from many of the consortia that attended the meeting, including USDA-ARS, USAID, IGGC, Centre for Tropical Livestock Genetics and Health (CTLGH), and others. Progress reports on CBBP in South Africa managed and funded by ARC and the University of KwaZulu-Natal, Malawi coordinated by Lilongwe University of Agriculture and Natural Resources (LUANR), and Uganda overseen by NARO were provided by representatives of those projects. The program also featured breakout sessions and follow-up discussions that focused on: the long-term sustainability of CBBP; capacity building in African membership countries; technical shortcomings; and research needs. Great interest was shown in expanding the CBBP model to additional member countries and much discussion centered on a continuity strategy for CBBP to become sustainable.

## 3 *de novo* goat genome assembly

From the very start of the efforts that led to the goat improvement program, constructing a *de novo* assembly of the goat genome was the highest priority objective under the project funded by the USAID. The highly fragmented nature of short-read assemblies, which were common at the time, fundamentally limited the reliability of genomic analyses. The hundreds of thousand gaps present in these genomes had deleterious effects on gene annotation, regulatory network analysis, association studies, and more. A group of researchers led by Wen Wang at the Beijing Genomics Institute was already building an assembly of the goat genome from short sequencing reads (Dong et al., 2013), so we requested access to their raw data to attempt a re-assembly using a long-read strategy. The leaders of that consortium declined to make that data available, so, our group felt it was necessary to develop an independent assembly of the goat genome.

**FIGURE 9:**
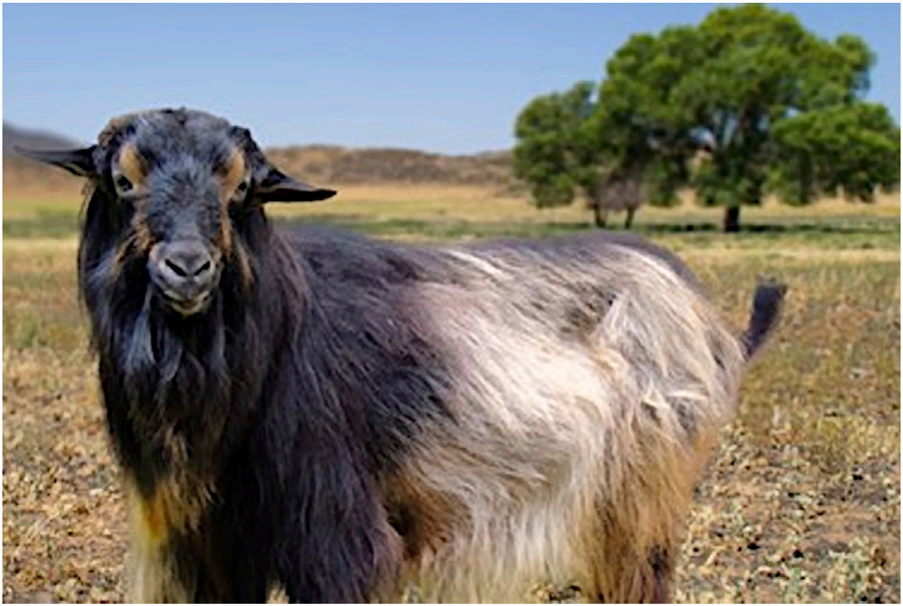
Papadum - The genome sequencing goat

Brian Sayre at Virginia State University led the effort to select the animal that was to be the donor of tissues used to build the genome assembly. Sequencing commenced with selection of a highly inbred male goat, “Papadum” (Figure 9). Assembling a genome is a complex problem that is further complicated in diploid organisms by the presence of both maternal and paternal chromosomes. Choosing an inbred individual minimized those haplotypic differences and simplified the assembly process. Papadum was an inbred member of an inbred breed, the San Clemente. This breed originated from San Clemente Island off the coast of San Diego, California. Because the San Clemente goats were confined to an individual island, they *inter se* mated, increasing levels of inbreeding. Tissue and DNA were sent to The US Meat Animal Research Center in Clay Center, Nebraska and the Animal Genomics and Improvement Laboratory (AGIL) at the Beltsville Agricultural Research Center for processing.

Previous work had shown that a mixture of sequencing reads from long- and short-read instruments improved the completeness of genome assemblies (Dalloul et al., 2010). But, sequencing reads from long read platforms resulted in an even more complete coverage of a genome (Pendleton et al., 2015). Eventually, multiple technologies would be combined to generate a genome assembly. These included Pacific Biosciences high error-rate long-read sequencing for assembly, BioNano optical mapping, and Hi-C, a genome-wide chromatin conformation capture protocol using proximity ligation, for scaffolding, and Illumina short-read sequencing for increasing the base accuracy of the assembly. The complementarity of these technologies led to dramatic improvements in genome assemblies (Bickhart et al., 2017; Worley, 2017). Nature published a Milestone collection for the 20th anniversary of the human genome sequence in 2021 with the goat assembly named as one of the 18 papers chosen as “milestones.” Quoting that article (LaFlamme, 2021), “The domestic goat genome ARS1 created a new standard for *de novo* assemblies of complex genomes.” The detailed description of the improvements made can be found in that publication (Bickhart et al., 2017). The quality of the assembly, ARS1, was described as Golden (Worley, 2017) and Platinum (LaFlamme, 2021).

## 4 Community-based breeding programs (CBBPs)

Community-based breeding programs (CBBPs) can have a positive impact on the local economy by helping farmers improve genetics and productivity that can in turn, lead to increased income for farmers and greater access to markets for their products. Additionally, by developing local capacity and expertise in breeding and management, these programs can help create jobs and support economic development in the community. In addition to working with farmers directly, a successful CBBP also engages the support of local government and community officials by educating them on the CBBP potential economic gains made possible with improved animal genetics. CBBPs are essentially systems that involve local communities to collaboratively and collectively develop breeding objectives that are applied across a combined communal herd (Sölkner et al., 1998; Haile et al., 2011; Wurzinger et al., 2011). The implementation approach for these CBBPs followed similar steps as those demonstrated with sheep in Ethiopia (Haile et al., 2011).

### 4.1 Negative selection

Farmers in developing countries are often under economic pressures to make short-term choices for economic gain that can negatively impact the overall genetics of their herd. This phenomenon is known as negative selection. For example, negative selection arises from removal of superior (i.e., larger) males from the breeding population through sales at earlier ages of these faster growing bucks to fetch higher market prices rather than retaining them for breeding (Gizaw et al., 2014). The long-term genetic impact of this short-sighted decision on the herd, is to leave slower growing males as the breeding males in the community flocks, perpetuating inferior genetics. CBBP training programs provide information to farmers on basic animal breeding strategies, the impacts of negative selection, and the importance of following breeding objectives. Together, these steps can lead them to select breeding bucks that meet their stated breeding objectives. For increased rate of growth, CBBP farmers find that in just a few generations all of their bucks are of the fast-growing type. AGIN CBBP programs provide farmers with the information and tools they need to identify their breeding objectives and select the very best young breeding bucks in the project, to keep them retained and available to the community.

### 4.2 Participatory approach

The participatory approach embedded in CBBP fosters the development of community-level capacity, engenders buy-in, and cultivates ownership among local farmers. This approach significantly reduces the likelihood of reverting to familiar, traditional breeding practices, including negative selection, once the programs conclude. By actively involving the farmers in the process of enhancing herd management and establishing dependable record-keeping systems, they acquire a sense of ownership, thus ensuring sustained progress beyond the program’s duration. In a CBBP, using local personnel to collect and manage animal production records is prioritized over relying on centralized support. The involvement of a local technician or enumerator plays an important role by providing a conduit to encourage communication between the farmers and the researchers during early stages of the CBBP. Key to the AGIN effort was training local doctoral students to become CBBP experts in their own countries to ensure sustainability of this little utilized, yet successful approach for livestock genetic enhancement in developing countries.

### 4.3 Locally adapted

Furthermore, CBBP often involves the use of locally adapted breeds that are better suited to the environmental conditions and farming practices of the area. This focus on local adaptation usually enhances herd resilience and adaptability, thus increasing food and nutrition security for the community. In contrast to historical, centralized breeding programs that introduced non-local or foreign breeds, use of local breeds allows farmers to see the potential, and ultimately the superiority of their locally-adapted animals. This realization can foster a point of pride among farmers.

Overall, CBBPs are an effective and sustainable approach to improving the genetic quality and productivity of livestock in developing countries. By involving local communities and building local capacity, these programs can promote sustainable practices and create lasting benefits for the farmers and their communities.

### 4.4 Steps to establish a successful CBBP

1. Identify the community: Identify and engage with the community to be involved in the program. This group should include a diverse group of individuals, including the appropriate local or regional officials who will offer support or champion the CBBP, along with the farmers, ranchers, and other community members who have an interest and role in breeding and raising livestock. Inclusion of women among the community members and farmers is critical to supporting and elevating families.
2. Define the breeding objectives: Once the community has been identified, it is critical to assess the breeding priorities. This process may include identifying the breeds or types of animals that are most in demand, as well as the specific breeding goals and objectives identified by the producers.
3. Develop selection strategy: Based on breeding objectives, a selection process is needed. This strategy may include ranking criteria [e.g., mass selection, index selection, or BLUP (Van Vleck, 1993)], mating strategies (e.g., buck management), or inbreeding management.
4. Implement the program: Once the breeding plan has been developed, it is time to implement the program. This may involve training local community members as enumerators, applying ear tags, obtaining tissue samples for DNA extraction, application of best practices for breeding and raising animals, as well as providing necessary resources or equipment.
5. Monitor and evaluate: It is important to continually monitor and evaluate the progress of the CBBP. This may include tracking the number and quality of animals that are produced. Based on the results, adjustments to the breeding plan may be necessary.
6. Create a sustainable system: Finally, it is essential to create a sustainable system for the CBBP. This includes establishing a system of record-keeping and data collection, providing ongoing training and support, establishment of legal breeder cooperatives, and encouraging community members to take ownership of the program.

### 4.5 Some key features of CBBP

• Involvement of local communities: Communities are actively engaged in the planning, implementation, and management of the program. This helps to ensure that the program is tailored to the specific needs and priorities of the community.
• Focus on genetic improvement: The program may involve the use of a range of breeding strategies, from basic recordkeeping and mass selection to artificial insemination, or genetic testing. This process can lead to increased productivity and better health of the animals.
• Promoting sustainable practices: CBBPs may also focus on promoting sustainability in livestock management, such as reducing the use of inputs like feed and water and reducing the environmental impact of the production system.
• Support for small-scale farmers: CBBPs often target smallholder farmers and pastoralists, often women, who may not have the resources or expertise to improve the genetics of their animals on their own. The program provides them with the necessary support and resources to do so.
• Another feature of CBBPs is that farmers pool their herds with those of other producers in their communities. This creates bigger and more diverse gene pools, enabling them to maintain genetic diversity and enhance selection opportunities.

### 4.6 Pilot CBBP projects

Uganda and Malawi were chosen to host pilot AGIN CBBP projects. Both countries have a high proportion of households that own and receive substantial portions of income from goats. Two locations were selected in each country with two communities per location chosen (a total of 8 sites). In Uganda, the final locations selected were Nakapiripit and Hoima. In Nakapiripit, two communities raising Small East African goats in a communal grazing system were chosen. In Hoima, Mubende goats are raised in two production systems, crop-livestock (tethering) and communal grazing. One community for each system was selected. In Malawi, communities within the Magoti extension planning area (EPA) and Zombwe EPA were selected. Small East African goats are found in both areas. Farmers in Magoti EPA practice communal grazing while those in Zombwe EPA favor tethering. In total, we monitored CBBPs in 5 communal grazing sites and 3 crop-livestock systems.

#### 4.6.1 Uganda

In Uganda, nearly 40% of households own goats, and all but 1% of those are indigenous. The Ugandan team introduced the CBBP concept and shared experiences with research stakeholders including AGIN partners and determined the best locations to initiate the Ugandan CBBPs - Katakwi and Nakapiripit (Small East African) and Masindi and Hoima (Mubende goat breed). They held meetings with district veterinarians, extension workers, and farmers and conducted field site visits. The characteristics of the sites follow.

##### 4.6.1.1 Katakwi

The principal breed represented in Katakwi is the Small East Africa goat, and there was generally negative attitude to indigenous goat breeds. Tethering is used by most farmers, and as a result there is limited mixing of flocks. The selection objectives include perceived breed purity, body size, and goat color. Castration has not been practiced in the past. There were limited farmer groups active in this region. The selling of the best performing males (i.e., negative selection) was common practice because they earned a better price in the market.

##### 4.6.1.2 Nakapiripit

While the Small East African goat was also the most common breed in Nakapiripit, the goats were tended using shared grazing resources. Households typically stay together using a communal “kraal,” a traditional African village of huts, typically enclosed by a fence. Selection goals include increased body size, twinning/triplet ability, and disease tolerance. Negative selection was practiced in Nakapiripit. The use of male selection through castration was practiced but not common. Improvement of productivity was a high priority here, but introduction of new breeds were not successful in prior experiences, so improvement of indigenous goats was important for local communities.

##### 4.6.1.3 Masindi

Mubende and the Small East African goat are both popular breeds of goats in Masindi. There are both crop-livestock production systems with 3-6 goats in each herd and pastoral-grazing systems with much larger herds (∼60 goats). Neither of these systems mixed herds. This community was characterized by poor breeding and management skills as well as a reluctance to work together in groups. This community has recently recognized the economic importance of goats.

##### 4.6.1.4 Hoima

The Mubende breed of goat dominates in Hoima, and there is a growing interest in goat production. The overall population of goats has grown. As in Masindi, there are both modest sized (∼5–6 goats) crop-livestock production herds and pastoral-grazing systems with much larger herds (∼50–300 goats in each herd). The farmers in Hoima had a good working knowledge and understanding of management practices: castration, disease control, genetics and reproduction. The selection objectives combined twining, increased birth weight, rapid growth, and large size. There was a group of strong active farmers (∼50 members) working with Zonal Agricultural Research Organization. There was active sharing of bucks (free for members; 0.80 USD per breeding for non-members). These farmers had strong attachments to goats for economic importance, however there were some challenges in retaining good bucks for enough time to impact genetic improvement.

The final sites selected were Nakapiripit consisting of two communities that used communal grazing production system falls under communal grazing, and Hoima with one communal grazing and one tethering system. Pre-printed ear tags were used to track the animals, and a full time PhD student was engaged in the project via BOKU, and the AGIN CBBP expert there who has successfully implemented CBBPs with native graduate students in several countries.

#### 4.6.2 Malawi

In Malawi, the percent contribution of livestock to household income ranged from 17% to nearly 60% in the Shire Valley. Additionally, goats contribute more income to households, especially female headed households. Negative selection in a subsistence culture contributed to declines seen in livestock production. The CBBP model offered an opportunity to improve animal productivity and animal genetic resource (AnGR) conservation. Twenty-six stakeholder participants attended the organizational meetings, and the meetings concluded with the value of the CBBP being recognized and supported by key organizations. Three potential CBBP sites were considered by the Malawi team:

##### 4.6.2.1 Magoti

Magoti Extension Planning Area (EPA) in Shire Valley Agricultural Development Division (ADD). These are communities that are dependent on livestock. They are also in regions that will have significant impact from climate change.

##### 4.6.2.2 Zombwe

Zombwe EPA in Mzuzu ADD. These are communities that are dependent on both crops and livestock, although this region has a strong culture and tradition of keeping livestock.

##### 4.6.2.3 Mitundu, Mkwinda and Chilaza

Mitundu, Mkwinda, and Chilaza EPA in Lilongwe ADD. These communities are primarily crop producers with a secondary dependence on livestock. This region provides proximity to research institutions (Lilongwe University of Agriculture and Natural Resources (LUANAR) and Chitedze Agricultural Research Station). Farmers had an existing rapport with livestock outreach programs (e.g., community dairying and indigenous chickens).

Due to the expressed support in the stakeholder meetings by government and non-governmental organizations groups, all three sites were selected. Farmers that met with the team understood the concept of the breeding program. They clearly appreciated the existing problem of negative selection. All three communities expressed a desire to participate. The markets in these areas for goat products are mainly for meat and are used directly within the farmer’s household or for ceremonies. Additionally, products could be marketed at various selling points or trading centers and are now found in some retail shops.

The CBBP model was scaled out to two other districts: Neno (Lisungwi EPA) and Salima (Matenje EPA). Additionally, some non-governmental organizations adopted the model and implemented it in three other districts: Dowa (Mvera EPA), Kasungu (Lisasadzi EPA) and Mzimba North (Bwengu EPA).

### 4.7 The future looks bright

#### 4.7.1 Ethiopia

Ethiopia CBBP started in 2009 with four populations (Afar, Bonga, Horro, and Menz) representing different production systems and involving 8 communities of about 500 households owning about 8,000 sheep. These pilot CBBPs have since expanded to include more than 150 communities. Though implemented at a pilot scale in Ethiopia, the CBBPs have resulted in quantifiable genetic gains and impacted the livelihoods of rural communities (Haile et al., 2020). CBBPs need to scale up to impact on the lives of larger communities. To this end, a methodological framework for scaling of CBBPs was developed (Mueller et al., 2019). AGIN supported scaling of goat CBBPs in Konso, Ethiopia and more than 2000 households were covered through this scheme. The Ethiopian government has identified CBBP as the strategy of choice and several scaling initiatives are being supported in Ethiopia through various projects.

#### 4.7.2 Burkina Faso

The objectives of this project were to:

1. Establish CBBPs for smallholder goats in two sites in Burkina Faso to genetically improve unselected indigenous goat breeds.
2. Explore the possibility of using unique DNA tools and genotype data to complement phenotype data.

One site of the CBBP implementation was the province of Namentenga located in the transition area between the Sahelian and Sudanian agro-ecological area. The second site was the province of Poni (Zone B) in the southwest of the country belonging to Sudanian agro-ecological area. Breeding systems in these areas are sedentary agropastoral system and transhumant pastoral system. Farmers are largely illiterate, with men slightly outnumbered by women.

The flock size is small (∼15), and bucks are selected based mainly on body size, coat color, and temperament. Does are selected based on body size, twinning ability, mothering ability, coat color, and, kidding frequency.

The project resulted in the implementation of 6 CBBPs at different sites with the involvement of all stakeholders. The participants universally appreciated the project. The results are quite encouraging and constitute assets for the implementation of programs on a larger scale. However, the management of selected breeding bucks and their sharing must be addressed within the communities. The results of the study already show that the management of bucks in a community grazing context is very tricky because they are not easy to control. Bucks are sometimes found in neighboring herds in search of does in heat and in some cases these bucks are not found. This phenomenon would explain the low number of bucks in some locations where owners never find them. Rather than lose their valuable animals, some farmers prefer to sell their goats at an early age.

## 5 Partnerships and leveraging

Collaboration has been a hallmark of this project. At the first organizational meeting of this group, we invited researchers and outreach professionals from a broad range of countries and organizations from within Africa and abroad. This heterogeneous group of collaborators generated the name African Goat Improvement Network (AGIN).

A highly collaborative effort was embraced for collecting biological samples and for genotyping. At the outset, the Feed the Future project received a “Greater Good Initiative” from Illumina, Inc., that represented the genotyping costs for about 400 animals. In addition, the Food and Agricultural Organization of the U.N. (FAO) solicited proposals to fund sample collection in four African countries, specifically excluding countries already well sampled by AGIN collaborators. The four countries that submitted proposals that were funded were: Egypt, Madagascar, Mali, and Tanzania. In addition, through collaborations, we had genotypes shared by Iowa State University (goats from Egypt), Catholic University in Italy (improved lines of Italian meat and dairy goats), Virginia State University (U.S. meat goat breeds and candidates for genome sequencing goat), University of Sao Paulo and Embrapa in Brazil (tropically adapted Brazilian goats), AgResearch in New Zealand (South African Boer goats), and the Agricultural Research Council (ARC) in South Africa (South African production and local goats). In total, over 4,000 goats have been sampled from 22 countries world-wide.

The first and most critical partnership that enabled these efforts was the one established by the Norman Borlaug Commemorative Research Initiative, a collaborative research effort between USDA-ARS and USAID. The goat improvement project has been well funded by the Feed the Future program in USAID and championed by several USAID (Max Rothschild, Lindsay Parish, Elaine Grings, and Saharah Moon Chapotin) and ARS (Eileen Herrara and Irlene Santos) leaders. Additional support has also been provided through the ARS Office of International Research Programs. At the time, one of their full-time employees, Jennifer Woodward-Greene, obtained her Ph.D. degree in bioinformatics, and her thesis project has contributed the field sampling protocol and phenotype prediction algorithms for characterizing morphometric measures of goats.

Despite the generous financial support, the funds were always tight, in part because genomics research is inherently expensive. Considering this situation, the funding from the Feed the Future program was highly leveraged to maximize the impact of these funds.

Another key partnership established was one with Johann “Hans” Sölkner at the University of Natural Resources and Life Sciences (Universität für Bodenkultur—“BOKU”) in Vienna, Austria. Hans has had a long and successful history of international development, including a true leadership in the development of CBBP in smallholder application. BOKU has played a critical role in our efforts to support graduate training and capacity building.

The first agreement established to support this project was done so as a direct result of the first meeting held on the campus of the International Livestock Research Institute (ILRI) in Nairobi, Kenya to support training and capacity building in bioinformatics. As part of this agreement, an ILRI scientist, Denis Mujibi, spent 6 weeks at the Bovine Functional Genomics Laboratory in Beltsville, Maryland working with USDA-ARS staff. ILRI also hosted the AGIN III meeting at its Addis Ababa, Ethiopia campus.

The engagement of three organizations was essential to establishing the CBBP: In Uganda, CBBP implementation is being facilitated by the National Livestock Resources Research Institute (NaLIRRI) under the umbrella of the National Agricultural Research Organization (NARO) and in Malawi, Lilongwe University of Agriculture and Natural Resources (LUANR). These organizations were the “boots on the ground” partners in the efforts to establish and grow CBBP in Africa.

### 5.1 International Goat Genome Consortium (IGGC)

The broad goal of the IGGC is to increase the knowledge of the goat genome and use that knowledge to answer important biological questions leading to expanded goat production around the world. The IGGC website is at www.goatgenome.org. The group formed in March 2010 with several initiatives: the generation of the first goat assembly, CHIR_1.0, led by Wen Wang at the Beijing Genomics Institute (Dong et al., 2013) and the design the first goat SNP chip led by Gwenola Tosser-Klopp at INRAE (Tosser-Klopp et al., 2014). The group, led by Gwenola Tosser-Klopp at the Institut National de Recherche pour l’Agriculture, l’alimentation et l’Environnement (INRAE, formerly INRA) in Toulouse, France, holds regularly scheduled communication meetings and coordinates goat workshops held annually at Plant and Animal Genome meetings. The AGIN group has interacted with this group, keeping them informed about genome assembly status and inviting them to AGIN meetings.

### 5.2 The AdaptMap project

It became clear that the best outcome for small holders was to identify those genomic regions important in stabilizing goat sustainability to parasites and drought. The optimal approach would be to compare for selective sweeps across global goat populations. Therefore, our project has joined forces with the IGGC and two EU consortia: 3SR—Sustainable Solutions for Small Ruminants and NextGen projects to form the AdaptMap project. Leveraging this partnership now aligns three goat genomics projects under one common goal—to understand diversity in goats for increased food production.

The AdaptMap project, led by Alessandra Stella from Istituto di Biologia e Biotecnologia Agaria in Lodi, Italy, is an international effort developed to improve coordination among otherwise independent projects for genotyping, sequencing and phenotyping of goat breeds. The aim is to explore diversity of breeds and populations around the world by using traditional and novel approaches. Since its inception, the centralized collection of genomic and phenotypic data from 15 projects on a total of 33 countries has started. Multiple actions have been undertaken to standardize genotypic and phenotypic data from different sources. These groups cover all aspects of the goat genome: i) the improvement of genome assembly; ii) genome annotation; iii) enhancement to the existing SNP genotyping platform; iv) the selection of a parentage and identity SNP panel; v) comparative genomics (with other ruminants); vi) integration and standardization of phenotypic data; vii) population genetics analyses and population history (domestication reconstruction); viii) landscape genomics; and, ix) breeding and genetic improvement. Working groups coordinated by leading scientists have been identified and several have completed their efforts (Bertolini et al., 2018; Colli et al., 2018; Stella et al., 2018).

An agreement was initiated to formalize a partnership with the AdaptMap Consortium and is intended to facilitate goat data sharing globally and encourage coordination and collaboration in characterizing the extensive variety of represented goat populations. The ultimate objective of AdaptMap is to enhance genetic improvement by understanding the adaptation of goats to diverse constraints. The efforts will result in a wide sampling of existing genetic diversity representing Africa as well as related non-African populations. This increased sampling will potentially increase power to detect signatures of selection, in addition to extending the training on phenotyping goats using the African Goat Improvement Network image collection protocol (AGIN-ICP).

### 5.3 VarGoats project

The VarGoats project has as a long-term goal to sequence over 1,000 goat genomes. The scientific objective is to identify variants in goat genomes associated with domestication and adaptation. Currently, the project has described a dataset of 1,159 goats, including over 250 individuals collected by AGIN (Denoyelle et al., 2021). The VarGoats website is located at www.goatgenome.org/vargoats.html. The VarGoats project was made possible by a call for large scale DNA sequencing projects by France Genomique. The data has been made available to VarGoats participants and data analysis is being performed in working groups (∼60 international scientific participants), most of them already created in the AdaptMap program.

### 5.4 The United Nations Food and Agricultural Organization (FAO)

The FAO recognizes animal genetics as one of “the pillars in livestock development,” with characterization, conservation, and genetic improvement representing three critical components of this pillar. In addition, characterization is a critical initial step in proper management of animal genetic resources (AnGR) to inform breeding programs and conservation decisions. FAO serves as the secretariat of the Intergovernmental Technical Working Group on Animal Genetic Resources for Food and Agriculture, which is a representative group of FAO member countries that advises on actions to be undertaken to improve the management of livestock genetic diversity. Paul Boettcher serves as the Secretary of the Working Group and has played a key role as an international coordinator of conservation of AnGR. Consequently, FAO supports the work of groups like AGIN to facilitate phenotypic and genomic characterization activities. The FAO also supports data collection and sharing through the Domestic Animal Diversity Information System (DAD-IS), a global database of AnGR to provide a data repository and a resource for sharing that data online. The FAO objective for this work is to achieve sustainable management of land, water, and genetic resources and improved responses to global environmental challenges affecting food and agriculture. With the assistance from donors, FAO has supported direct funding of AnGR characterization such as AGIN as part of its effort to achieve this outcome. This collaboration is a 3-way partnership, with FAO conducting field sample collection and compiling phenotypic data and pedigrees, USDA-ARS is providing equipment and guidance for sampling as well as DNA extraction and genotyping, and the AdaptMap consortium is providing data analysis and interpretation. Additionally, ASARECA is providing technical advice. A November 2013 call for proposals to implement the AGIN sample collection (genotype and phenotype) method yielded 14 proposals from 12 countries. Four of these were selected: Egypt (Egypt National Research Center), Madagascar (Département de Recherches Zootechniques et Vétérinaires du Centre National de Recherche Appliquée au Développement Rural), Mali (Programme Petits Ruminants, Institut d’Economie Rurale), and Tanzania (Tanzania Veterinary Laboratory Agency - Tanzania Livestock Research Institutes and Districts).

### 5.5 Additional partnerships

Partnerships were also formed with the Association for Strengthening Agricultural Research in Eastern and Central Africa (ASARECA) in Entebbe, Uganda; the National Biotechnology Development Agency in Abuja, Nigeria; Agricultural Research Council (ARC) of South Africa; and the International Center for Agricultural Research in the Dry Areas (ICARDA) in Ethiopia, the Center for Tropical Livestock Genetics and Health (CTLGH) in Edinburgh, Scotland and Nairobi, Kenya; São Paulo State University in Araçatuba, Brazil; Università Cattolica del S. Cuore in Piacenza, Italy; the Egyptian Ministry of Agriculture; Virginia State University in Petersburg, Virginia, USA; and the Iowa State University Global Food Security Consortium in Ames, Iowa, United States. Many of these partnerships were created to facilitate tissue and data collection and enable the broadest representation of goats for genetic and genomic comparison.

## 6 Training and professional development

### 6.1 Training for AGIN image collection protocol

A system was developed by USDA-ARS scientists within AGIN to enable collection of body measurements and other physical features from digital images and image analysis tools. This protocol was formalized and shared through AGIN’s AdaptMap partnership for international utilization. As part of the development and training component of this project, about a dozen phenotyping kits have been distributed and training has been conducted.

Johann Sölkner, Solomon Abegaz, and Tesfaye Getachew (BOKU), Denis Mujibi and Absolomon Kihara (ILRI), Brian Sayre (Virginia State University), Clet Masiga (ASARECA) were trained to use the data collection protocol with a hands-on training. Farai Muchadeyi (ARC) and Christopher Mukasa (Ahmadu Bello University) were trained remotely using online tools. Sampling kits have been provided to researchers associated with ILRI, ASERECA, BOKU, and ARC. Over time, a large number of AGIN members received training on the AGIN image collection protocol (AGIN-ICP).

Researchers from Ethiopia, Italy, Kenya, Malawi, Mozambique, Nigeria, Rwanda, South Africa, Tanzania, Uganda, The United Sates, and Zimbabwe were trained to obtain digital images and to collect body measures. Coordination of sample collection was led by ASARECA and ARS. At that time, phenotypes (digital images and body measurements) and tissue samples were collected from more than 1,800 goats in 10 countries (7 African countries).

### 6.2 High-school students

Goat field sampling data and geographic information system information was contributed by Brian Sayre at Virginia State University to share with 14 high school students in the Appomattox Regional Governor’s School of Art and Technology in Petersburg, Virginia, United States. Students used the GIS information to mine data related to natural resources, weather patterns, economic indicators, and cultural practices in each specific region.

### 6.3 Undergraduate students

In preparation for phylogenetic analyses, Heather Huson, Cornell University, had undergrad research assistants, Mary Beth Hannon and John Nystrom, update maps with sampling sites. They have identified nearest weather data stations to those sites. Processing raw body measurement data from Ethiopia, Kenya, and all ASARECA sites to determine average, maximum, minimum and standard deviation on all phenotyping data was initiated. Measurements were categorized by breed and country as well.

Heather Huson at Cornell University developed an international internship experience for undergraduate students to work with the Agricultural Research Council (ARC) in South Africa to collect field data at CBBP and process samples in the laboratory.

### 6.4 Graduate students

Jennifer Woodward-Greene completed her dissertation and defense and earned her doctorate in May 2016 from her research activities associated with the AGIN project. She continued this work with AGIN, which involved development and refinement of algorithms to extract phenotypic data from digital photos. One of the phenotypes included animal body measurements (height, length, girth) to predict animal body weight when scales are not available due to cost or convenience. Other phenotypes included FAMACHA anemia score, tooth age/health assessment, and coat color/pattern identification. Her work uses digital images that can be taken with a common cell phone, and development of the software for automated, “born-digital,” on-farm, collection of animal records. This work with AGIN provided a once-in-a-lifetime experience to lead a multi-national effort to develop the AGIN Image Collection Protocol (AGIN-ICP), [see companion paper (Woodward-Greene et al, 2023) describing how the AGIN CBBP model was used as a capacity development platform]. To process the collected images, she developed the PreciseEdge Image Segmentation Algorithm (Woodward-Greene et al., 2022) that isolates and collects (extracts) animal measurement from AGIN-ICP collected images. The manuscript is in process to describe the user-friendly software she developed to deploy the algorithm and related tools for researchers or farmers to collect digital phenotypes *in situ*.

Visits to USDA by Priscilla Ramadimetja Mohlatlole and Keabetswe Tebogo Ncube to build additional capacity with our South African partners as part of a larger collaborative effort by ARS and ARC. They were South African doctoral students under the mentorship of Farai Muchadeyi (ARC) and Edgar Dzomba (University of KwaZulu-Natal) and were selected in 2016 to conduct research at the USDA, ARS Animal Genomics Improvement Lab (AGIL) at the Beltsville Agricultural Research Center in Beltsville, Maryland (Curt Van Tassell’s lab). Ms. Mohlatlole was in her second year of a PhD in Animal Breeding at the ARC and University of KwaZulu-Natal, and Ms. Ncube had recently completed her MSc at the University of South Africa and was a first-year PhD student with ARC and the University of KwaZulu-Natal. Their planned research while at ARS was directly applicable to the USDA—USAID Feed the Future Livestock Improvement Project with aims to achieve objectives set by ARS, ARC and USAID related to the project. Ms Ncube earned her doctorate degree in April 2020. Her PhD research focused on differential gene expression studies to investigate the genetics of meat and carcass quality traits in South African indigenous goats. The project drew from the principles of AGIN-CBBPs that enabled her to monitor goats on-farm within the CBBP households of Pella village in South Africa and conduct a set of transcriptome experiments using goats from Pella village and the ARC experimental farms. The time spent at AGIL gave her access to computational resources and bioinformatics expertise to help her through the analysis.

Farai Muchadeyi, (AGIN partner) and graduate student Khanyisile Mdladla—Hadebe visited the laboratory of Heather Huson at Cornell University in July 2015 to expand their knowledge in genomic population structure and admixture analysis. This work contributed to Ms. Mdladla’s doctoral research and used local data from goats sampled in South Africa as part of ARC’s collaboration with AGIN and AdaptMap. Ms Khanyi was in her second year as a PhD student at the University of KwaZulu-Natal, South Africa under the mentorship of Farai Muchadeyi (ARC) and Edgar Dzomba (University of KwaZulu-Natal).

The goat improvement project supported the research and training of doctoral student, Wilson Nandolo, who worked in Malawi and Ugandan village breeding programs for sustainable genetic improvement. Mr. Nandolo worked along with researchers at the Lilongwe University of Agriculture and Natural Resources (LUANAR) in Malawi, and the National Agricultural Research Organization (NARO) in Uganda. He was trained and mentored by Hans Sölkner. The existing agreement with BOKU supported the CBBP in Malawi and Uganda, as well as Mr. Nandolo’s visit to the AGIL for training in genomic sequencing and analyses techniques. He worked on copy cumber variation analyses in the goat related to various traits of interest and provided support in the phenotype software development to collect phenotypic data on coat color and pattern from images.

The goat improvement project supported the work of doctoral student Doreen Lamuno. Ms. Lamuno, much like Mr. Nandolo, worked in Malawi and Uganda CBBP along with LUANAR and NARO while mentored by Hans Sölkner, with an emphasis on the systematic evaluation to provide guidance for an assessment of the performance, outputs, and associated impacts of CBBP.

Wilson Kaumbata was the third African PhD student attending BOKU who was added to the CBBP project. Mr. Kaumbata led the follow-on assessment of the CBBPs for the two established breeding communities. This development was timely, as the CBBPs were firmly established and progressing well. This work contributed to national goat breeding strategies, exploring the economic and social impacts of the breeding programs, and developing and testing approaches to ensure the village breeding program models employed in Uganda and Malawi could be scaled up (i.e., assess/develop technology transfer applicability) for application in other communities.

### 6.5 Sabatical

Denis Mujibi from International Livestock Research Institute was hosted by Curt Van Tassell and Tad Sonstegard for training in population genomics, computational genomics of next-generation sequence data, and genetics and breeding.

Brian Sayre from Virginia State University was hosted by Curt Van Tassell on a Faculty Research Fellowships for Capacity Building at 1890 Land-Grant Universities. The research projects centered on the use of goat genomics and genetics to strengthen smallholder livelihoods and communities in Africa. Additionally, our research had a focus on identifying adaptability traits in goats to improve sustainable food production for the future.

Clet Wandui Masiga from Association for Strengthening Agricultural Research in Eastern and Central Africa (ASARECA) was hosted at Cornell University on a sabbatical visit and worked with Heather Huson to learn about population genetic methods using AGIN samples and data.

## Abbreviations

3SR, Sustainable Solutions for Small Ruminants; ADD, Agricultural Development Division; AGIL, Animal Genomics and Improvement Laboratory (ars.usda.gov/agil); AGIN, African Goat Improvement Network; AGIN-ICP, AGIN image collection protocol; AnGR, animal genetic resources; ARC, South African Agricultural Research Council (arc.agric.za); ARS, Agricultural Research Service (ars.usda.gov); ASARECA, Association for Strengthening Agricultural Research in Eastern and Central Africa (asareca.org); BMGF, Bill and Melinda Gates Foundation (gatesfoundation.org); BOKU, Universität für Bodenkultur Wien (boku.ac.at/en/) (University of Natural Resources and Life Sciences, Vienna); CBBP, community-based breeding programs; CTLGH, Centre for Tropical Livestock Genetics and Health (www.ctlgh.org); DAD-IS, Domestic Animal Diversity Information System (https://www.fao.org/dad-is/en/); DNA, deoxyribonucleic acid; EPA, Extension Planning Area; FAO, Food and Agriculture Organization of the United Nations (fao.org); ICARDA, International Center for Agricultural Research in the Dry Areas (icarda.org); IGGC, International Goat Genome Consortium (goatgenome.org); ILRI, International Livestock Research Institute (ilri.org); INRAE, Institut National de Recherche pour l’Agriculture, l’alimentation et l’Environnement (inrae.fr/en); LUANR, Lilongwe University of Agriculture and Natural Resources (luanar.ac.mw); NaLIRRI, National Livestock Resources Research Institute (nalirri.or.ug); NARO, Ugandan National Agricultural Research Organisation (naro.go.ug); OIREC, Office of International Research Engagement and Cooperation (ars.usda.gov/oirec); OIRP, Office of International Research Programs; SNP, single nucleotide polymorphisms; USAID, United States Agency for International Development (usaid.gov); USDA, United States Department of Agriculture (usda.gov). See abbreviations table (Supplemental Materials).
