# Supplementary material for: The African Goat Improvement Network: a scientific group empowering smallholder farmers
Source: Front Genet. 2023 Aug 29;14:1183240. doi: 10.3389/fgene.2023.1183240 (PMC10497955; doi:10.3389/fgene.2023.1183240)
Supplement: Supplementary file 1 [file Table1.docx]

# Supplementary Table 1: Acronyms and abbreviations:

3SR – Sustainable Solutions for Small Ruminants

ADD – Agricultural Development Division

AGIL – Animal Genomics and Improvement Laboratory ([ars.usda.gov/agil](https://www.ars.usda.gov/northeast-area/beltsville-md-barc/beltsville-agricultural-research-center/agil/))

AGIN – African Goat Improvement Network

AGIN-ICP – AGIN image collection protocol

AnGR – animal genetic resources

ARC – South African Agricultural Research Council ([arc.agric.za](https://usdagcc-my.sharepoint.com/personal/curt_vantassell_usda_gov/Documents/arc.agric.za))

ARS – Agricultural Research Service ([ars.usda.gov](https://www.ars.usda.gov/))

ASARECA – Association for Strengthening Agricultural Research in Eastern and Central Africa ([asareca.org](https://asareca.org/))

BMGF – Bill and Melinda Gates Foundation ([gatesfoundation.org](https://www.gatesfoundation.org/))

BOKU – Universität für Bodenkultur Wien ([boku.ac.at/en/](https://boku.ac.at/en/)) (University of Natural Resources and Life Sciences, Vienna)

CBBP – community-based breeding programs

CTLGH – Centre for Tropical Livestock Genetics and Health ([www.ctlgh.org)](http://www.ctlgh.org))

DAD-IS – Domestic Animal Diversity Information System (<https://www.fao.org/dad-is/en/>)

DNA – deoxyribonucleic acid

EPA – Extension Planning Area

FAO – Food and Agriculture Organization of the United Nations ([fao.org](https://www.fao.org/))

ICARDA – International Center for Agricultural Research in the Dry Areas ([icarda.org](http://www.icarda.org/))

IGGC – International Goat Genome Consortium ([goatgenome.org](https://www.goatgenome.org/))

ILRI – International Livestock Research Institute ([ilri.org](https://www.ilri.org/))

INRAE – Institut National de Recherche pour l’Agriculture, l’alimentation et l’Environnement ([inrae.fr/en](https://www.inrae.fr/en))

LUANR – Lilongwe University of Agriculture and Natural Resources ([luanar.ac.mw](https://www.luanar.ac.mw/) )

NaLIRRI – National Livestock Resources Research Institute ([nalirri.or.ug](https://nalirri.or.ug/))

NARO – Ugandan National Agricultural Research Organisation ([naro.go.ug](https://naro.go.ug/))

OIREC – Office of International Research Engagement and Cooperation ([ars.usda.gov/oirec](https://www.ars.usda.gov/office-of-international-research-engagement-and-cooperation/office-of-international-research-engagement-and-cooperation/))

OIRP – Office of International Research Programs

SNP – single nucleotide polymorphisms

USAID – United States Agency for International Development ([usaid.gov](https://www.usaid.gov/))

USDA – United States Department of Agriculture ([usda.gov](https://www.usda.gov/))
